# A Pedestrian Dead Reckoning Method for Head-Mounted Sensors

**DOI:** 10.3390/s20216349

**Published:** 2020-11-07

**Authors:** Xinyu Hou, Jeroen Bergmann

**Affiliations:** Natural Interactions Lab, Department of Engineering Science, Institute of Biomedical Engineering, University of Oxford, Oxford OX1 2JD, UK; xinyu.hou@eng.ox.ac.uk

**Keywords:** inertial measurement unit, navigation, smart glasses, wearable sensors, virtual reality

## Abstract

Pedestrian dead reckoning (PDR) plays an important role in modern life, including localisation and navigation if a Global Positioning System (GPS) is not available. Most previous PDR methods adopted foot-mounted sensors. However, humans have evolved to keep the head steady in space when the body is moving in order to stabilise the visual field. This indicates that sensors that are placed on the head might provide a more suitable alternative for real-world tracking. Emerging wearable technologies that are connected to the head also makes this a growing field of interest. Head-mounted equipment, such as glasses, are already ubiquitous in everyday life. Whilst other wearable gear, such as helmets, masks, or mouthguards, are becoming increasingly more common. Thus, an accurate PDR method that is specifically designed for head-mounted sensors is needed. It could have various applications in sports, emergency rescue, smart home, etc. In this paper, a new PDR method is introduced for head mounted sensors and compared to two established methods. The data were collected by sensors that were placed on glasses and embedded into a mouthguard. The results show that the newly proposed method outperforms the other two techniques in terms of accuracy, with the new method producing an average end-to-end error of 0.88 m and total distance error of 2.10%.

## 1. Introduction

In recent years, human position tracking technology has been rapidly developing and it has drastically changed everyday life by offering location information, even during complex scenarios. Location Based Services (LBS) can help to provide accurate tracking of human position and this has now been widely adopted in a range of different fields. Local spatial data are often being leveraged to provide specific services to users. It can offer continuous monitoring, location-specific information when the user is nearby a point of interest or even provide specialistic services upon request. Users can benefit from this technology by being able to find specific destinations and it has been suggested that LBS is becoming one of the most important sources of revenue for the wireless communications industry [1].

The Global Navigation Satellite System (GNSS) is a widely used position tracking method, but it is not a suitable solution inside confined spaces, due to an often rapid deterioration of the signal strength when these spaces are entered. Nonetheless, position tracking for indoor environments is playing an ever increasing role in human navigation, which is further propelled by the rise of smart devices. An example of this is the application of indoor tracking within the healthcare sector. This technology can be used to give hospitals an understanding of how best to allocate clinical staff or assist with patient monitoring. Therefore, it is immensely valuable to find suitable techniques that can accurately localize humans indoors. A lot of varied methods have been developed by researchers and they frequently leverage information regarding the Received Signal Strength (RSS) of Wireless Local Area Network (WLAN) [2]/Bluetooth low energy (BLE) beacons [3]/ultra wideband (UWB) [4], radio frequency identification (RFID) [5], ultrasound [6], and other modalities. Unfortunately, many of these methods rely on external aiding signals, information, or infrastructure, and, thus, they are not applicable in scenarios where the signals are severely affected or when there is no specific infrastructure available. The construction of new infrastructures to facilitate indoor tracking also comes at an added cost, as it can be expensive to setup and maintain these frameworks.

On the contrary, self-contained position tracking does not rely on any infrastructure and they are often based on Pedestrian Dead Reckoning (PDR) methods. These approaches can utilize low-cost on-body sensors, including Inertial Measurement Units (IMUs), accelerometers, gyroscopes, compasses, barometers, magnetometers, anemometers, or on-body cameras, to name just a few, for examples, [7,8,9,10]. The information from these sensors is subsequently used to calculate an estimate of the current position, thus providing infrastructureless applicability to the users. For examples, it is suitable during environmental emergencies, as the surroundings might be frequently changing. It also works well under smoky and dim-lit conditions, which makes it ideal for firefighting or rescue operations that could encounter similar environments. It can also be adopted in the sports. The trajectories of athletes could be recorded indoor or outdoor, which could be analysed by coaches for performance improvement. The trajectory length that the athletes have covered during the basketball or football game can also reflect the amount of exercise, and fatigue levels could be estimated, which could be applied by the coach, as a training tool, in order to create a competitive advantage [11] and avoid injuries.

A recent systematic literature review [12] showed that the majority of PDR papers for wearable sensors apply sensors positioned on the foot. This is closely followed by publications that use smartphone based sensors. The waist is the third most researched option, which is followed by the leg and upper torso. There were only two published papers identified that looked at PDR for head-mounted sensing systems. Most studies adopted foot-mounted sensors, as this location makes it is easier to detect specific gait features that are reoccuring during walking. This gait information can be utilized by subsequently applying the Zero Velocity Update (ZUPT) or Zero Angular Rate Update (ZARU) techniques. The ZUPT and ZARU allow for a reduction in the long-term accumulation of errors. However, when potential users within a healthcare setting were asked where they would like to wear sensor technologies, placement on the foot was rarely mentioned (only 2% of the time this location came-up) [13]. It was also shown that small, discreet, and unobtrusive systems were preferred, with many people referring back to everyday objects. This would indicate that objects, such as glasses, might be more acceptable for monitoring in an everyday environment, whilst the applicability of smart mouthguards or helmets might be more appropriate within the (contact) sports community.

Glasses are already a requisite to people who need to correct for certain visual impairments and it is estimated that there are 1406 million people with near-sightedness globally (22.9% of the world population). This number is predicted to rise to 4758 million (49.8% of the world population) by 2050 [14]. Besides vision correction, people also wear glasses for protection from ultraviolet light or blue light, or just to accessorize. There are already several smart glasses products on the market, such as Google Glasses, Vuzix Blade, Epson Moverio BT-300, Solos, and Everysight Raptor. The field of smart glasses also links in well with the growing interest in providing tracking in virtual reality (VR) environments without the need of any other technology except what is integrated into the VR headset.

Mouthguards are very essential in contact sports to protect athletes. It was estimated that there are 40 million mouthguards sold in the United States each year [15]. With the increasing participation in contact sports, the consumption of mouthguards will continue to climb. Studies on smart mouthguards with acoustic sensors for breathing frequency detection [16] has shown the feasibility of sensor-embedded mouthguards. Smart mouthguards may be the future of the mouthguard industry, as it combines physical protection with relevant information for the sport community [17].

Moreover, in humans the stability of the visual field is essential for efficient motor control. The ability to keep the head steady in space allows for control of movement during locomotor tasks [18]. Therefore, the placement of sensors on the head would provide the very suitable location, as the whole body is working on stabilizing that particular segment of the system. The head is also where the vestibular system is located, which acts as an inertial guidance system in vertebrates. Placing the artificial positional tracking system near the biological one seems to be a fitting approach.

For head-mounted solutions, Hasan and Mishuk proposed an IMU sensor fusion algorithm, which utilizes data that were collected by a three-axis accelerometer and three-axis gyroscope that were embedded into smart glasses [19]. The glasses were worn by a subject and steps were detected by applying peak detection of the accelerometer norm. The linear model in (7) is used to obtain a step length estimation. An Extended Kalman Filter (EKF) is used to then fuse the accelerometer and gyroscope data in order to determine the heading direction. A Kalman Filter (KF) is a recursive Bayesian filter, which is known to be an optimal filter for Gaussian linear systems. KF uses a series of measurements that are observed over time (containing statistical noise and other inaccuracies) in order to produce estimates of unknown variables. These estimates tend to be more accurate than those that are based on a single measurement alone. An output from the filter is obtained by estimating the joint probability distribution over the variables for each given time frame. The EKF is the non-linear version of the KF, where the state transition and observation matrices are Jacobian matrices of non-linear measurement model equations. The state vector that was used in the aforementioned study was:

$$x_{t}={\left[\theta_{x}\;\theta_{y}\;\theta_{z}\;\omega_{x}\;\omega_{y}\;\omega_{z}\right]}^{T}$$

and the measurement vector was:

$$y_{t}={\left[\omega_{x}\;\omega_{y}\;\omega_{z}\;a_{x}\;a_{y}\;a_{z}\right]}^{T}$$

where θ represents the Euler angle (${}^{\circ }$), ω is angular rate (${}^{\circ }$/s), and *a* is acceleration (ms${}^{-2}$) on each axis. The result that was found by [19] showed that the sensor fusion positioning technique accomplished an average error between estimated and real position of 2 m in the most complex case, when the monitoring time frame was less than 2 min.

Zhu et al. [20] utilized a hybrid step length model and a new azimuth estimation method for PDR. The steps were detected by the peak detection or the positive zero crossing detection algorithm. The hybrid step length model is given in (1),

(1) $$l=\left(a\cdot \left(r_{lf}\cdot f+r_{lv}\cdot v\right)+b\right)\cdot \sqrt[{4}]{a_{max}-a_{min}}$$

where *a* and *b* are coefficients, *f* is the walking frequency (Hz), *v* is the accelerations’ variance. $a_{max}$ and $a_{min}$ (ms${}^{-2}$) are the maximum and minimum acceleration in one step, $r_{lf}$ is the Pearson correlation coefficient between the step length and the walking frequency, and $r_{lv}$ is the Pearson correlation coefficient between the step length and accelerations’ variance.

(2) $$r_{lf}=\frac{\sum_{i=1}^{N}\left(l_{i}-\bar{l}\right)\left(f_{i}-\bar{f}\right)}{\sqrt{\sum_{i=1}^{N}{\left(l_{i}-\bar{l}\right)}^{2}}\cdot \sqrt{\sum_{i=1}^{N}{\left(f_{i}-\bar{f}\right)}^{2}}}$$

(3) $$r_{lv}=\frac{\sum_{i=1}^{N}\left(l_{i}-\bar{l}\right)\left(v_{i}-\bar{v}\right)}{\sqrt{\sum_{i=1}^{N}{\left(l_{i}-\bar{l}\right)}^{2}}\cdot \sqrt{\sum_{i=1}^{N}{\left(v_{i}-\bar{v}\right)}^{2}}}$$

The heading information was generated from the hybrid value of the azimuth that was estimated by gyroscopes and magnetometers:

(4) $$\varphi_{hyb}=k_{gyr}*\varphi_{gyr}+k_{mag}*\varphi_{mag}$$

where $\varphi_{gyr}$ and $\varphi_{mag}$ (${}^{\circ }$) are the heading angles that were estimated by gyroscope and magnetometer, $k_{gyr}$ and $k_{mag}$ are coefficients, and $\varphi_{hyb}$ (${}^{\circ }$) is the hybrid heading estimation. In their study, they found a maximum error of 1.44 m and a mean error of 0.62 m.

We propose exploring the performance of these two PDR methods (which are specifically for head mounted sensors) [19,20] and a novel algorithm that was based on Peak detection, Mahony algorithm, and Weinberg step length model. These three methods will be compared and the average end-to-end error, as well as total distance error will be recorded. The proposed PDR method has a higher accuracy than the two currently available methods. The performance is consistent across different sensors and placements, which verifies its robustness. This method provides a suitable approach across various hardware platforms and, thus, supports the potential creation of truly innovative smart head-mounted systems in the long-term.

## 2. Methods

The proposed techniques belongs to the field of Step-and-Heading Systems (SHSs). SHSs output a series of step vectors by detecting each step of the pedestrian, estimating the length and direction of it, and finally integrating every step to obtain a complete trajectory. The next position of the pedestrian could then be estimated in (5) when the current position after $k_{th}$ step $\left(x_{k},y_{k}\right)$ is known, where $l_{k+1}$ (in m) and $\varphi_{k+1}$ (in ${}^{\circ }$) represent the step length and forward direction of the next step.

(5) $$\begin{array}{c} x_{k+1}=x_{k}+l_{k+1}*\sin\left(\varphi_{k+1}\right)\\ y_{k+1}=y_{k}+l_{k+1}*\cos\left(\varphi_{k+1}\right) \end{array}$$

### 2.1. Step Detection

Accurate step detection is a primary requirement for precise position estimation with PDR. A peak detection method was used in this study to detect a step at a heel strike. First, the norm of accelerometer signal was calculated by (6), where $A_{x}$, $A_{y}$, $A_{z}$ (ms${}^{-2}$) represent accelerations on three axes.

(6) $$Accel_{norm}=\sqrt{A_{x}^{2}+A_{y}^{2}+A_{z}^{2}}$$

**Figure 1:**
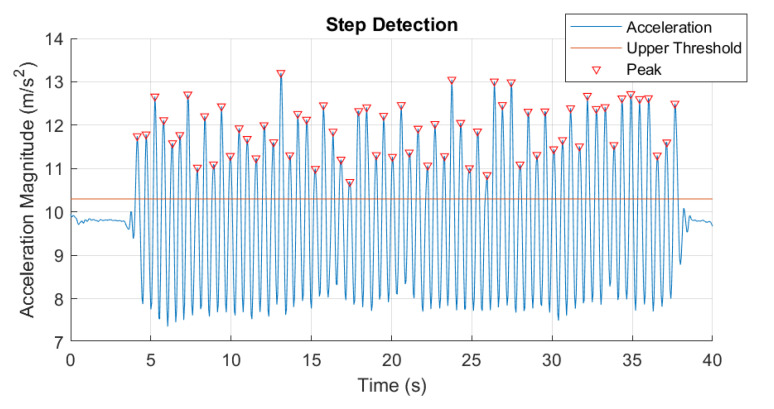
Step detection result.

Subsequently, the accelerometer norm was filtered by a first-order low pass filter (LPF) with a cut-off frequency set to 2 Hz to eliminate any high frequency noise, which should be sufficient for capturing the walking motion of most users. The filtered signal peaks that crosses the minimum threshold are detected as a step. The period between two adjacent peaks represent the process of the center of gravity moving from the lowest point to the highest point and back again, which corresponds to a single step when walking. Therefore, the peak detection method can be applied in order to detect each steps (see Figure 1).

### 2.2. Step Length Estimation

Step length is the distance between the point of initial contact of one foot and the point of initial contact of the opposite foot. In normal gait, right and left step lengths are relatively similar. However, step length does vary between people according to height, gender, age, physical condition, etc. An inertial navigation systems (INSs) calculates distance by the double integration of the acceleration signal. Yet, the integration error due to noise, bias, and other disturbances is not negligible and it can increase rather quickly. There are various algorithms designed for correction or compensation on these errors, such as zero velocity update (ZUPT), which utilizes the stance and swing phase during walking. This implies that ZUPT is not suitable for PDR with head mounted sensors. Machine learning methods were also normally used in step length estimation, such as using stacked autoencoders [21], LSTM, and denoising autoencoders [22], etc. There are other model-based step length estimators, which propose equations in order to capture the relationship between step length and other step characteristics, such as step frequency, maximum or minimum acceleration or acceleration variation. For example, Do et al. [23] estimated the horizontal displacement while using vertical acceleration, which is based on the double integration of the vertical acceleration, followed by using the inverted pendulum model. The most commonly used models consist of the linear model [24], Weinberg model [25], Kim model [26], Scarlett model [27], and Shin model [28].

1. Linear model represents the linear relationship between step length and walking frequency *f* (Hz).
  (7) step_length=a·f+b
2. Weinberg method utilizes the difference of the vertical acceleration values within a step.
  (8) $$step\_length=k\cdot \sqrt[{4}]{a_{max}-a_{min}}$$
3. Kim method is only based on the average acceleration within a step.
  (9) $$step\_length=k\cdot \sqrt[{3}]{\frac{\sum_{i=1}^{N}\left|a_{i}\right|}{N}}$$
4. Scarlett method eliminates the spring effect of the human gait by using minimum, maximum and average acceleration.
  (10) $$step\_length=k\cdot \frac{\frac{\sum_{i=1}^{N}\left|a_{i}\right|}{N}-a_{min}}{a_{max}-a_{min}}$$
5. In Shin model, not only the step frequency but also the variance during that step is involved. So it is more precise than the frequency singly related model listed before.
  (11) step_length=a·f+b·v+c

where *a*, *b*, *c*, and *k* are coefficients, $a_{max}$ and $a_{min}$ (ms${}^{-2}$) are the maximum and minimum accelerations in one step, *N* is the number of samples in one step, *v* is variance of accelerometer signal in one step, and $a_{i}$ stands for the accelerometer signal at time *i*.

The above methods were tested on single user gait data (walking in a straight path). The preliminary results presented in Table 1 show that the Weinberg, Kim, and Shin model have the best performance with the head-mounted sensors in this study. When considering the speed of calculation, the Weinberg model was finally chosen as the step length estimator in this study. The application of a single step length estimator across the PDR algorithms allowed for a more unbiased assessment of the performance within this study.

### 2.3. Heading Estimation

Mahony et al. [29] proposed an explicit complementary filter, which considers the problem of obtaining good attitude estimates from measurements that were obtained from typical low-cost inertial measurement units. This algorithm only requires accelerometer and gyro outputs in order to estimate heading direction. In this study, a quaternion-based derivation of the explicit complementary filter was adopted as the heading estimator.

The explicit complementary filter in terms of unit quaternion representation can be expressed in (12), where $\Omega_{y}$ is the biased measure of angular velocity by gyroscope, $k_{P}$ is the proportional gain, and $k_{I}$ is the integral gain, p(Ω)=(0,Ω) is a pure quaternion, $\hat{b}$ denotes gyro bias, $\omega_{\mathrm{mes}}$ is a correction term, and can be thought of as a non-linear approximation of the error.

(12) $$\begin{array}{cc} \omega_{\mathrm{mes}} & =-\mathrm{vex}\left(\sum_{i=1}^{n}\frac{k_{i}}{2}\left(v_{i}{\hat{v_{i}}}^{T}-{\hat{v}}_{i}v_{i}^{T}\right)\right)\\ \dot{\hat{q}} & =\frac{1}{2}\hat{q}⊗p\left(\Omega_{y}-\hat{b}+k_{P}\omega_{\mathrm{mes}}\right)\\ \dot{\hat{b}} & =-k_{I}\omega_{\mathrm{mes}} \end{array}$$

When implementing it, the accelerometer data (showing the direction of gravity) are used as a reference. First, the estimated direction of gravity is calculated by the unit quaternion. The orthogonal matrix corresponding to a rotation by the unit quaternion q=a+bi+cj+dk (with |q|=1), when post-multiplying with a column vector, is given by:

(13) $$\begin{array}{ccc} \; & R=\; & \left(\begin{array}{ccc} a^{2}+b^{2}-c^{2}-d^{2} & 2bc-2ad & 2bd+2ac\\ 2bc+2ad & a^{2}-b^{2}+c^{2}-d^{2} & 2cd-2ab\\ 2bd-2ac & 2cd+2ab & a^{2}-b^{2}-c^{2}+d^{2} \end{array}\right) \end{array}$$

Subsequently, the estimated gravity direction is:

(14) $$\begin{array}{cc} {\hat{v}}_{i} & ={\hat{R}}^{T}{\left[\begin{array}{ccc} 0 & 0 & 1 \end{array}\right]}^{T}\\ \; & ={\left[\begin{array}{ccc} 2bd-2ac & 2cd+2ab & a^{2}-b^{2}-c^{2}+d^{2} \end{array}\right]}^{T} \end{array}$$

The measured gravity direction is:

(15) $$v_{i}=\frac{a}{\left|a\right|}$$

The error is given as the cross product between estimated gravity direction and measured direction:

(16) $$e_{i}=v_{i}\times \hat{v_{i}}$$

In this sample period, the quaternion rate of change is:

(17) $${\dot{\hat{q}}}_{i}=\frac{1}{2}{\hat{q}}_{i-1}⊗p\left(\Omega_{i}+k_{P}\cdot e_{i}+k_{I}\cdot \sum_{i=1}^{N}e_{i}\right)$$

The estimated quaternion in this sample period is:

(18) $${\hat{q}}_{i}^{\prime }={\hat{q}}_{i-1}+{\dot{\hat{q}}}_{i}\cdot t$$

where *t* is the length of sample period. Finally, the normalized quaternion will be outputted:

(19) $${\hat{q}}_{i}=\frac{{\hat{q}}_{i}^{\prime }}{|{\hat{q}}_{i}^{\prime }|}={\left[\begin{array}{cccc} q_{0} & q_{1} & q_{2} & q_{3} \end{array}\right]}^{T}$$

After calculating this for each sample period, a series of estimated quaternions is generated. In order to obtain the heading angle, each quaternion is subsequently transformed into an Euler angle:

(20) $$\left[\begin{array}{c} \phi \\ \theta \\ \psi \end{array}\right]=\left[\begin{array}{cc} atan2(2\left(q_{0}q_{1}+q_{2}q_{3}\right), & 1-2(q_{1}^{2}+q_{2}^{2}))\\ \mathrm{asin}(2(q_{0}q_{2} & -\;\;q_{1}q_{3}))\\ atan2(2\left(q_{0}q_{3}+q_{1}q_{2}\right), & 1-2(q_{2}^{2}+q_{3}^{2})) \end{array}\right]$$

The Mahony algorithm can also fuse the magnetometer data into the calculation. Yet, our proposed method did not include any magnetometer data into the final estimation due to the unpredictable magnetic interference and the noise in the low-cost sensors.

## 3. Experimental Conditions and Results

### 3.1. Hardware Description

The sensor that was placed on the glasses and the mouthguard is the SensorTile Microcontroller Unit (MCU) module (STEVAL-STLCS01V1), which includes a low-power three-dimensional (3D) accelerometer and 3D gyroscope (LSM6DSM), ultra-low power 3D magnetometer (LSM303AGR), Bluetooth low energy network processor (BlueNRG-MS), 32-bit ultra-low-power MCU with Cortex®M4F (STM32L476JG), and a 100 mAh lithium-Ion polymer battery. Data were collected at 20 Hz and transferred to a mobile phone by Bluetooth.

**Figure 2:**
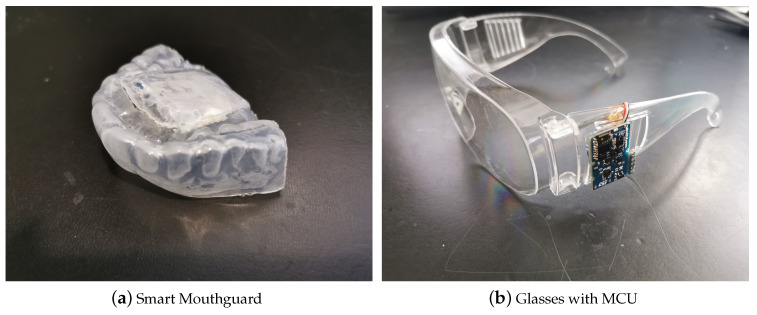
Devices used in this study.

The MCU was attached to the glasses and then embedded into a mouthguard. The mouthguard was made out of two Ethylene-vinyl acetate (EVA) layers with 0.6 mm thickness, which were thermoformed whlie using the upper teeth cast of the subject. The MCU was embedded between the EVA sheets and placed near the hard palate of the oral cavity (Figure 2a). The MCU was also attached on the legs of a pair of glasses with sa ensor located near the left temple (Figure 2b). Both of the placements provided a rigid arrangement that ensured the sensors precisely followed the head motions.

### 3.2. Data Collection

Three healthy adults voluntarily participated in this study, including one female volunteer and two male volunteers. In [12], the majority of PDR studies (66.2%) had only one participant, which made the results somewhat limited. This paper had three subjects and data were collected four times with each device.

**Figure 3:**
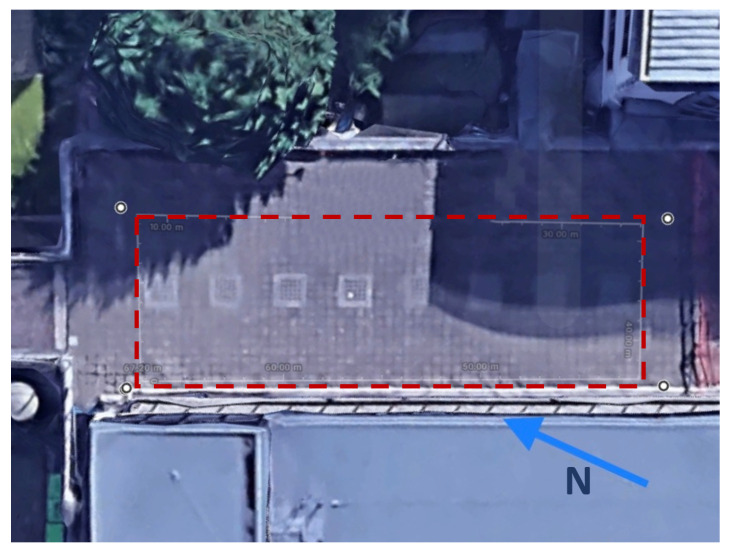
Top view of the data collection trajectory that was set out for each subject. The red dotted line shows the trajectory subjects were asked to walk.

The tests were undertaken on a hard outdoor surface during a clear day. The red dotted line that is shown in Figure 3 is the walking trajectory used the tests. The set out path formed a rectangle with an approximate length of 25.5 m and width of 8.5 m. The satellite image that is shown in Figure 3 is from Google Map.

The subjects were asked to face the walking direction and stand still for 5 s at the beginning of each data collection trial. They were then requested to walk at a normal and constant speed whilst keeping their head facing the walking direction. When the subject reached the end of the trajectory, they were asked to stop walking and stand still again for another 5 s. Specific information was given regarding walking speed, as it is known that small changes in instructions given could change the test outcome [30]. Ethical approval was obtained from the University and this experiment was part of a larger study (R43470/RE001).

All data analysis was conducted in MATLAB (R2020a, Mathworks, Natick, MA, USA).

### 3.3. Results

**Figure 4:**
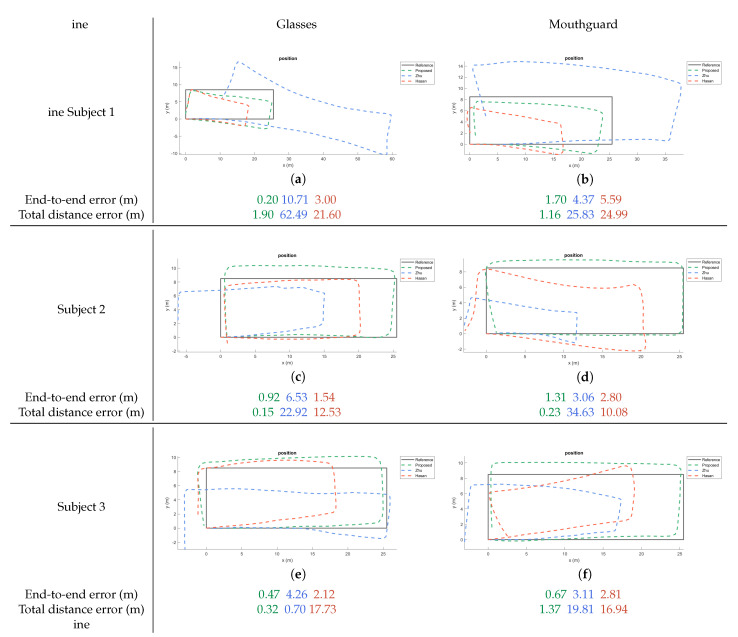
Estimated trajectories for the three methods plotted against the ground-truth. One of the measurements (randomly selected) of each subject is shown for each placement. The end-to-end error and total distance error (m) are shown in the sequence of proposed, Zhu’s, and Hasan’s algorithm.

Figures in Figure 4 show the estimated trajectories with the glasses and mouthguard by Hasan’s, Zhu’s and the newly proposed algorithm. The proposed method generated trajectories that are closer to the ground-truth trajectory when compared to the other two methods.

**Figure 5:**
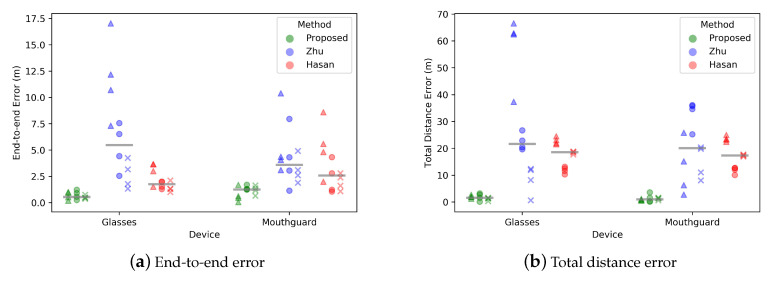
Errors across all subjects for each condition. Horizontal lines represent median values. A triangle is used to represent data from subject 1, a circle is given for subject 2 and data from subject 3 is shown as a cross.

The end-to-end error represents the distance between start point and end point of the estimated trajectory. The total distance error is the absolute difference between real and estimated trajectory lengths. The mean end-to-end error of Hasan’s method is 2.62 m and Zhu reached 5.41 m, while the new method reached a mean end-to-end error of 0.88 m. Figure 5a shows that Zhu’s method has a larger variance of the error with different sensor layouts, while the proposed method has the smallest mean error and smallest variance for both sensor locations.

The novel method has a mean total walking distance error of 1.43 m, which is 2.10% of the total length. However, Hasan’s and Zhu’s methods have a relatively larger error rate of 25.97% and 36.36%, respectively. The total distance errors are shown in Figure 5b.

## 4. Discussion

**Figure 6:**
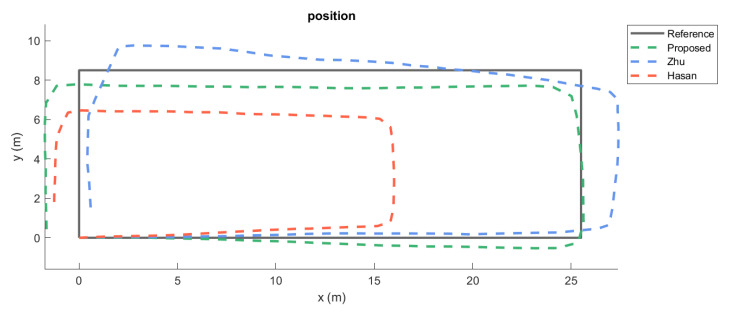
Estimated trajectories for the three methods plotted against the ground-truth for a head-mounted smart phone. The measurement is from one subject.

The results show that the end-to-end and total distance error was the lowest in terms of central tendency for the newly proposed method. Extra tests were carried out with a different set of sensor platform (smartphone) for provide a further preliminary cross-platform comparison. The data was collected using a Huawei P30 smartphone, which consists of three-axis accelerometer and gyroscope (ICM-20690) produced by TDK InvenSense, and three-axis magnetometer (AK09918) from Asahi Kasei Microdevices. The smartphone was placed on the cranial part of the head and was kept firmly in place while using adaptable straps. Only one subject was tested under this head-mounted smartphone condition. The three methods all performed comparatively well for the heading estimation under the smartphone condition (see Figure 6), which is likely due to the higher accuracy of the IMU in the smartphone as compared to the MCU. However, when a cheaper IMU is used (such as the one attached on the glasses and mouthguard), the results of Hasan’s method and Zhu’s method quickly perform with greater errors (Figure 4). The newly proposed method in this article seems rather robust (in terms of errors), even when the sensor platform changes, which indicates a more broader inclusion of hardware is possible and that the system is likely to perform relatively stable even under less optimum operational conditions.

For the heading estimation, the error of Zhu’s method was heavily influenced by sensor type and layout. The main reason for the lack of robustness in this algorithm is likely due to the fact that the noise from the sensors is not well compensated for. The hybrid heading estimation is simply the proportional sum of raw integration of gyroscope and magnetic direction, with the proportional gain being setted manually. Although, it sets a threshold for the gyroscope to start integration only when the Euclidean norm of the gyroscope data is above this threshold, the bias in gyroscopic data still remains and thus the integration error remains. The heading estimation from the magnetometer is also still an problem, because the geomagnetic field is not always uniform [31] and it can be easily disturbed by hard or soft magnetic interferences. In addition, the oscillation during walking creates non-negligible high frequency noise in the heading estimation.

Regarding the step length estimation, Hasan’s and Zhu’s method yield larger errors for a range of reasons. Hasan’s algorithm used a linear model with the coefficient values being adopted from another study, which obtained parameters from 4000 steps of 23 different people [24]. The linear regression based on this data would provide a general model and this model does not necessary scale well across individuals. Especially, if the conditions, subjects or instructions differ from the original database. Generating specific parameters for each individual is therefore a key factor to get more accurate step length estimations. As for the Zhu’s method, the step length estimation model combines information of step frequency, acceleration variance, as well as maximum and minimum accelerations in one step. This is a more scalable solution, which should yield more accurate estimates. Nonetheless, this method only performs well when the conditions and sensor placement are exactly the same between the step length estimation session and the subsequent tracking sessions. If changes occur between these sessions then the step length estimate might no longer be accurate enough under the new conditions. Even when the temperature or the sensor changes, the acceleration variance, maximum and minimum acceleration can start to vary, because of the changing noise or the different sensor properties [32]. This can lead to contrasting estimations, even when all other conditions remain the constant.

The attitude representation in Hasan’s method and in the proposed method of this study is notably different. The former uses Euler angles in the EKF, which consists of three rotation angles around three axes. The latter adopted quaternions within the Mahony algorithm. The Euler angles are more human understandable, but have several disadvantages. These include the ambiguity in the rotation sequence of axes and the possible occurrence of a gimbal lock which leads to the loss of one degree of freedom. In contrast, expressing rotations as unit quaternions has some advantages, such as: concatenating rotations is computationally faster and numerically more stable; extracting the angle and axis of rotation is simpler; interpolation is more straightforward and quaternions do not suffer from gimbal lock as Euler angles do [33]. Although the EKF with Euler angle in Hasan’s method performs well, it would be more stable if quaternions are used.

Hasan’s and the newly proposed method showed high accuracy in heading estimations, proving the effectiveness of EKF and Mahony’s algorithm in the attitude estimation. This is reflected by the fact that they are already one of the most popular algorithms in this area. In EKF, there are usually two parameters that need to be set and tuned, which are the process noise covariance matrix *Q* and measurement noise covariance matrix *R* [34]. In Mahony’s algorithm, there are also two parameters that need tuning: proportional gain $k_{P}$ and integral gain $k_{I}$. In both of these algorithms, the parameters are found by tuning until the best results are achieved. This is, of course, time consuming and there is no guarantee of optimality. More importantly, when the sensor position is changing, for example, from glasses to the mouthguard, the parameters need retuning. More research on automatic tuning of parameters will be useful, as it can help with the creation of the next generation of algorithms.

The processing of the recorded data in this study was done off-line. Nonetheless, many applications will need online PDR to provide real-time navigation or localisation. The PDR algorithms should be assessed for online implementation feasibility and preliminary estimates of running time provide some insights into this. The running time of each method is shown in Table 2. Hasan’s method takes much more time than the other two methods, because the Jacobian calculation in EKF is complex and time-consuming. The mean running time of one sample is 0.0661 s, which indicates that the sampling frequency will be lower than 15 Hz. The empirical run-time estimates are limited as algorithms are platform-independent and, for that reason, more theoretical assessment needs to take place to compare the complexity of these algorithms, which is beyond the scope of this paper.

The subjects were instructed to follow the reference path that was shown in Figure 4 and Figure 6. However, it is likely that there is some deviation from the ideal path, especially during turning, as the subjects were asked to walk in their own preferred manner. Nonetheless, all of the subjects always started and stopped in the same location. Caution needs to be taken regarding to the generalizability of the results. Different environmental conditions, such as pathways taken or surrounding objects, can yield different outcomes. A simple walking pattern (rectangle) was selected in this study in order to minimise any trial variability between subjects and sensor placement. It should be noted that most studies use only a single person in their testing [12] and that this study included multiple subjects. It will be useful for the field to further expand the number of subjects, as well as environmental conditions (e.g., walking surface) during experimental testing to create ever increasing levels of external validity.

The method that was presented in this paper was the best performing algorithm across the different sensor locations. This new technique offers an approach that can be generalized across sensors and smart head-mounted equipment, such as helmets, hats, glasses, mouthguard, dentures, or earphones. The ability to have a high-performing general algorithm for head-based systems can provide new opportunities for a range of industries, ranging from healthcare to entertainment.

The current experiment did not collect synchronised data from the different locations. Thus, any assessment of the relationship between these locations needs to take into account, the subject variability between trials. Nonetheless, the task at hand was relatively simple to complete and variability is likely to be limited within a subject. Information about how strongly these placements are correlated allows for further insight in terms of potential reciprocal nature of these systems. The Spearman’s rank correlation coefficient (rs) was calculated to assess this, as a Pearson Correlation is susceptible to outliners in small sample sizes. The coefficients were determined between the glasses and mouthguard condition for the proposed (rs = 0.4261, *p* = 0.0390), Zhu’s (rs = 0.6435, *p* = 0.0009) and Hasan’s (rs = 0.9920, *p* = 3.0338 $\times \;10^{21}$) method. It showed a strong correlation across all methods between the locations. This indicates that these systems can potentially act interchangeably. More work is needed in order to further confirm these preliminary findings regarding the association between different head-mounted sensor positions.

## 5. Conclusions

In this paper, we proposed a PDR method for head-mounted equipment. Peak detection was used to detect steps and heading estimation was carried out with the Mahony algorithm. The Weinberg model was implemented to obtain step length estimation. This proposed method was executed in MATLAB with another two methods for performance comparison. To evaluate the accuracy of three methods, we collected walking data on a rectangle trajectory while using low-cost IMUs on glasses and in a mouthguard, respectively. The result shows that the proposed method reached an average end-to-end error of 0.88 m and total walking distance error of 2.10%. A consistent performance was obtained for the novel algorithm across conditions indicating the potential robustness of the proposed method. This algorithm provides potential implementation into various hardware platforms, which can be translated into truly innovative smart head-mounted systems in the long-term.

## Figures and Tables

**Table 1:** Comparison of step length estimation algorithms (Real distance: 11.28 m).

Methods	Linear	Weinberg	Kim	Scarlett	Shin
Estimated distance (m)	8.86	11.11	11.23	10.08	11.12
Error (m)	2.41	0.16	0.05	1.20	0.15
Error rate	21.42%	1.48%	0.45%	10.67%	1.41%

**Table 2:** Running time of methods.

Methods	Hasan	Zhu	Proposed
Number of samples	701	701	701
Total time (s)	46.3434	0.2263	0.9543
Mean time (s)	0.0661	0.0003	0.0014
